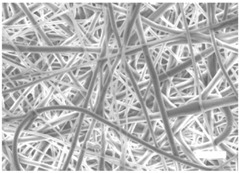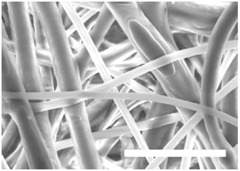# Electrospinning PCL Scaffolds Manufacture for Three-Dimensional Breast Cancer Cell Culture

**DOI:** 10.3390/polym9080328

**Published:** 2017-08-01

**Authors:** Marc Rabionet, Marc Yeste, Teresa Puig, Joaquim Ciurana

**Affiliations:** 1 New Therapeutic Targets Laboratory (TargetsLab)—Oncology Unit, Department of Medical Sciences, Faculty of Medicine, University of Girona, Emili Grahit 77, 17003 Girona, Spain; m.rabionet@udg.edu; 2 Product, Process and Production Engineering Research Group (GREP), Department of Mechanical Engineering and Industrial Construction, University of Girona, Maria Aurèlia Capmany 61, 17003 Girona, Spain; 3 Biotechnology of Animal and Human Reproduction (TechnoSperm), Department of Biology, Institute of Food and Agricultural Technology, University of Girona, Pic de Peguera 15, 17003 Girona, Spain; marc.yeste@udg.edu

**Keywords:** poly(ε-caprolactone), electrospinning, scaffolds, three-dimensional cell culture, triple negative breast cancer, breast cancer stem cells, mammospheres, aldehyde dehydrogenase

## Abstract

In vitro cell culture is traditionally performed within two-dimensional (2D) environments, providing a quick and cheap way to study cell properties in a laboratory. However, 2D systems differ from the in vivo environment and may not mimic the physiological cell behavior realistically. For instance, 2D culture models are thought to induce cancer stem cells (CSCs) differentiation, a rare cancer cell subpopulation responsible for tumor initiation and relapse. This fact hinders the development of therapeutic strategies for tumors with a high relapse percentage, such as triple negative breast cancer (TNBC). Thus, three-dimensional (3D) scaffolds have emerged as an attractive alternative to monolayer culture, simulating the extracellular matrix structure and maintaining the differentiation state of cells. In this work, scaffolds were fabricated through electrospinning different poly(ε-caprolactone)-acetone solutions. Poly(ε-caprolactone) (PCL) meshes were seeded with triple negative breast cancer (TNBC) cells and 15% PCL scaffolds displayed significantly (*p* < 0.05) higher cell proliferation and elongation than the other culture systems. Moreover, cells cultured on PCL scaffolds exhibited higher mammosphere forming capacity and aldehyde dehydrogenase activity than 2D-cultured cells, indicating a breast CSCs enrichment. These results prove the powerful capability of electrospinning technology in terms of poly(ε-caprolactone) nanofibers fabrication. In addition, this study has demonstrated that electrospun 15% PCL scaffolds are suitable tools to culture breast cancer cells in a more physiological way and to expand the niche of breast CSCs. In conclusion, three-dimensional cell culture using PCL scaffolds could be useful to study cancer stem cell behavior and may also trigger the development of new specific targets against such malignant subpopulation.

## 1. Introduction

Presently, in vitro cell culture represents a crucial tool to study cell behavior outside the organism. Most cell cultures are performed with a two-dimensional (2D) environment providing cheap and easy cell maintenance. A flat plastic surface is treated, obtaining adherent features to enable cell adhesion and proliferation. Therefore, these cells can only grow forming a monolayer, establishing interactions with surface and contiguous cells. Cells adopt a flattened morphology, which results in a modified membrane receptor polarity and cytoskeleton architecture. Different studies demonstrated that cell shape variations interfere with gene expression and protein synthesis regulation [1,2]. Consequently, the 2D cell culture model described differs from the physiological environment of living organisms. Body cells are embedded in the extracellular matrix (ECM), a three-dimensional (3D) complex constituted by fibrous proteins and molecules. This network structure provides a physical support for cell growth as well as playing a major role in cell regulation [3,4]. Cells can establish connections with their adjacent counterparts and with ECM fibrous mesh, thereby tending to adopt a more elongated morphology. This clear 3D physiological architecture contrasts with the lack of structure of two-dimensional cell culture. Hence, conclusions from in vitro monolayer cell culture experiments could be not applicable in terms of in vivo cell behavior, empowering the requirement of 3D models for cell culture.

Over recent years, various three-dimensional cell culture systems have emerged, differing in their composition, arrangement and final application. The models based on a solid, physical support are called scaffolds and are made up of a network of filaments mostly made by synthetic materials [5]. This 3D product can be manufactured by electrospinning technology using a high electric field. The polymer is dissolved and the solution is charged at high voltage. When electric force overcomes the surface tension, the polymer solution is pulled onto the target plate and the solvent is evaporated, collecting nanofibers which intersect each other [6]. The resultant architecture mimics the ECM fibrous assemblage and cultured cells are able to adopt a more in vivo shape. Obviously, all cell types possess distinct morphological characteristics which may lead to different cell culture support requirements. In this regard, it is worth noting that scaffold manufacturing techniques allow product customization, so that different process parameters, such as scaffold porosity, fiber diameter and microstructure, may be modified on the basis of the final application [7,8,9]. Poly(ε-caprolactone) (PCL) is a synthetic polymer that has been widely used to fabricate scaffolds, due to its viscoelastic and malleable properties, absence of isomers and low cost [10]. PCL may be processed with many technologies since it presents a low melting point around 60 °C and it is soluble in several solvents, such as chloroform, dichloromethane, benzene, acetone and dimethylformamide [11]. Moreover, PCL shows biocompatible properties, long-term biodegradability but bioresorbable [12], all these features making it a good candidate for biomedical and cell culture applications.

As previously explained, polymeric filaments offer physical support to cells to adhere and proliferate into the 3D structure. This fact enables cells to acquire a more elongated shape, close to physiological morphology, in contrast with the flatness adopted in monolayer cultures. Hence, the cancer research field is taking advantage of the three-dimensional cell culture model’s benefits. Over the last two decades, cancers have not been studied as an abnormal growth of a single cell type, as cells with distinct characteristics, such as normal cancer cells and, in minor proportion, cancer stem cells (CSCs) are found in a given tumor. While the CSCs subpopulation only represents a small percentage of tumor cells, they have been demonstrated to drive cell growth in a wide variety of cancer types including leukemia [13], brain [14,15], myeloma [16] and breast [17]. Therefore, CSCs possess tumorigenic features, among other specific characteristics, useful for their identification and isolation. This subset is capable of undergoing self-renewal and differentiating into non-stem cancer cells due to their stem properties. Moreover, CSCs are able to grow in suspension and proliferate forming spheres [14,18], and can be isolated due to an enhanced activity of the aldehyde dehydrogenase (ALDH) enzyme [19]. As expected, this subpopulation with stem characteristics plays a key role in cancer development and prognosis. A link between CSCs and tumor relapse after treatment and metastasis is proven [20] since they show relative high radio- [21] and chemoresistance [22]. This fact becomes relevant in some specific cancer types with an appreciable recurrence percentage, such as breast cancer. Concretely, triple negative breast cancer (TNBC) presents the highest proportion of tumor relapse with a value around 34% and the lowest mean time to local and distant recurrence when compared with other breast cancer types [23]. TNBC is characterized by the absence of breast cancer molecular biomarker amplification, so therapeutic targets against TNBC do not exist and patients are treated with general chemotherapy [24].

Breast cancer stem cells (BCSCs) could become a potential target for future treatments against TNBC. However, BCSCs in vitro culture encounters a number of difficulties. Cancer stem subpopulation represents a low percentage within the tumor [17,25] and two-dimensional cell culture induces its differentiation losing stem features [26]. Previous investigations demonstrated that three-dimensional cell culture maintained and expanded BCSCs subset when compared with 2D culture samples [27,28,29,30]. Therefore, the present sought to test the suitability of fabricated electrospun PCL scaffolds to provide a more suitable niche for BCSCs to grow. Scaffolds from two different polymer concentrations were tested to evaluate 3D culture suitability with triple negative breast cancer cells. Cell proliferation and morphology were evaluated on different culture days and finally, BCSCs were quantified to discern the scaffold’s culture effect. In agreement with literature, PCL scaffolds could be a useful tool to culture breast cancer cells in a more physiological way and expand the BCSCs subpopulation. Customizable methodologies such as electrospinning enable the production of distinct three-dimensional meshes concerning the cell of interest requests. Moreover, the BCSCs’ enrichment could facilitate their study and the development of specific treatments against this malignant subpopulation. BCSCs targeted treatments could replace aggressive procedures like chemotherapy and attack the highly recurrent tumors such as triple negative breast cancer.

## 2. Materials and Methods

### 2.1. Scaffolds Fabrication

Poly(ε-caprolactone) (PCL) and acetone were chosen as biopolymer and non-toxic solvent respectively, to manufacture the scaffolds. PCL 3 mm pellets with an average molecular weight of 80,000 g/mol (Sigma-Aldrich, St. Louis, MO, USA) were dissolved in acetone (PanReac AppliChem, Gatersleben, Germany). Two different concentrations of 7.5 and 15% *w/v* PCL were achieved under 40 °C and agitation using a magnetic stirrer. Scaffolds were fabricated with an electrospinning instrument (Spraybase, Dublin, Ireland). PCL solution was placed in a plastic syringe (BD Plastipak, Franklin Lakes, NJ, USA) connected to an 18 G needle emitter with an inner diameter of 0.8 mm. A fixed voltage of 7 kV was applied and a flow rate of 6 mL/h was established by the Syringe Pump Pro software (New Era Pump Systems, Farmingdale, NY, USA). The distance between the emitter and stationary collector was 15 cm. The electrospinning process was stopped when 10 or 5 mL of solution were ejected, for 7.5 and 15% PCL concentrations respectively. The meshes were then cut into squares with a scalpel.

### 2.2. Scanning Electron Microscopy Analysis

Microscopic characterization was performed through scanning electron microscopy (SEM; Zeiss, Oberkochen, Germany) after carbon coating. Scaffolds were imaged on the top and bottom to confirm fibre uniformity and Image J software (National Institutes of Health, Bethesda, MD, USA) was used for image analysis. Fibre diameter, surface porosity and pore area were calculated from the top and bottom sides to calculate the average value.

### 2.3. Cell Line

MDA–MB–231 triple negative breast cancer cell line was obtained from the American Type Culture Collection (ATCC; Rockville, MD, USA). Cells were routinely grown in Dulbecco’s Modified Eagle’s Medium (DMEM; Gibco, Waltham, MA, USA) supplemented with 10% fetal bovine serum (FBS), 1% l-glutamine, 1% sodium pyruvate, 50 U/mL penicillin/streptomycin (HyClone, Logan, UT, USA). Cells were kept at 37 °C and 5% CO_2_ atmosphere and culture medium was changed every 3 days.

### 2.4. Three-Dimensional Cell Seeding

PCL meshes were sterilized by immersion into 70% ethanol/water solution overnight, washed three times with PBS (Gibco, Waltham, MA, USA) and finally exposed to UV light for 30 min. Sterilized scaffolds were placed in non-adherent cell culture microplates (Sartstedt, Nümbrecht, Germany) and soaked in culture medium for 30 min at 37 °C before cell seeding to facilitate cell attachment. Corresponding cell density was prepared in a small volume of medium (50–100 μL). Cell suspension was pipetted drop by drop onto the scaffold centre. Then scaffolds were incubated for three hours at 37 °C and 5% CO_2_ atmosphere to allow cell attachment and after that incubation period, culture medium was added.

### 2.5. Cell Proliferation Assay

A suspension of 100 MDA–MB–231 cells per cm^2^ were seeded on adherent microplate wells (Sartstedt), 7.5% and 15% PCL scaffolds. Cell culture was maintained for 12 days. Every two days, samples were collected and 3-(4,5-dimethylthiazolyl-2)-2,5-diphenyltetrazolium bromide (MTT) assay was performed to quantify cell viability. Briefly, adherent wells and scaffolds were washed with PBS and meshes were put into new wells. Volumes of 1 mL DMEM and 100 μL MTT (Sigma-Aldrich, St. Louis, MO, USA) were added and samples were incubated for 150 min. In this test, only viable cells retain the ability of transforming yellow MTT into purple formazan crystals. After incubation, formazan crystals were dissolved with 1 mL DMSO (Sigma-Aldrich, St. Louis, MO, USA) under shaking. Four 100 μL aliquots from each well were pipetted into a 96-well plate and placed into a microplate reader (Bio-Rad, Hercules, CA, USA). Absorbance was measured at 570 nm. Culture medium of remaining samples was changed every two days.

### 2.6. Three-Dimensional Cell Culture

In order to evaluate the amount of BCSCs, MDA–MB–231 cells were cultured for 3, 6 and 12 days on scaffolds without passaging, changing the culture medium every three days. Considering cell growth kinetics of MDA–MD–231 cell line, 20,000, 8000 and 400 cells/cm^2^ were seeded for 3, 6 and 12 days of culture respectively, achieving a similar cell confluence at the end of each culturing period. Since cell confluence affects cell behaviour and metabolism, the final cell amount was fixed to avoid variations due to this effect. In the case of 2D samples, cells were cultured on monolayer for 3 days at a cell seeding density of 20,000 cells/cm^2^ in the same way as the scaffolds. Prior to the present work, cell line was grown routinely on two-dimensional plastics, thus only 3 days culture time was performed. Preceding experiments showed no differences in reference of cell behaviour between 2D cultured cells during 3, 6 and 12 days using the aforementioned initial cell densities (20,000, 8000 and 400 cells/cm^2^ respectively; data not shown).

### 2.7. Fluorescence Microscopy Analysis

Triple negative MDA–MB–231 cells were cultured on adherent coverslips (Sarstedt) and 7.5 and 15% PCL meshes, for 2D and 3D culture respectively. After the culture period, cells were washed with PBS and fixed with 4% paraformaldehyde (PFA; Sigma, St. Louis, MO, USA) for 20 min. To permeabilize the cells, coverslips and scaffolds were washed and 0.2% Triton X-100 (Sigma) was added for 10 min. Then samples were blocked with PBS containing 3% bovine serum albumin (BSA; Sigma) as a blocking buffer for 20 min. Cells were subsequently incubated at room temperature for 20 min with rhodamine-phalloidin (Cytoskeleton Inc., Denver, CO, USA) (1:200) to stain actin cytoskeleton and then with 4,6-diamidino-2-phenylindole (DAPI; BD Pharmingen, Franklin Lakes, NJ, USA) (1:1000) also at room temperature for 10 min to stain nuclei. Fluorescence was observed under a fluorescent microscope (Zeiss Axio Imager Microscope, Carl Zeiss, Göttingen, Germany) and a Nikon DS-Ri1 coupled camera (Nikon, Tokyo, Japan) was used to acquire all images. Camera settings (illumination intensity, quality, resolution and colour) were standardised for all photographs. Rhodamine-phalloidin (red) and DAPI (blue) fluorescence were captured and merged with Image J software (National Institutes of Health, Bethesda, MD, USA). This software was also used to calculate nuclear and cytoplasmic elongation factors. In brief, five cells of ten different images were randomly selected to measure the length and width of the nucleus and cytoplasm as shown in the following formula:

$$Nuclear/Cytoplasmic\;Elongation\;Factor=\;\frac{length\;nucleus/cytoplasma}{width\;nucleus/cytoplasma}\;\geq 1$$

### 2.8. Mammosphere-Forming Assay

Scaffolds were washed with PBS and put into new wells to collect only those cells attached to PCL filaments. Cells from 2D culture and scaffolds were detached with trypsin-EDTA (Cultek, Madrid, Spain) at 37 °C and 5% CO_2_ atmosphere. Afterwards, trypsinization cells were resuspended with DMEM/F12 medium (HyClon) containing the following supplements: B27 (Gibco, Waltham, MA, USA), EGF and FGF (20 ng/mL; Milteny Biotec, Bergisch Gladbach, Germany), 1% l-glutamine and 1% sodium pyruvate. A suspension of 2000 cells/well was seeded onto a 6-well, non-adherent cell culture microplate (Sarstedt) and incubated for 7 days at 37 °C and 5% CO_2_. After this period, spherical mammospheres bigger than 50 μm were counted. The equation described below was used to calculate the Mammosphere Forming Index (MFI) of each culture condition:

$$MFI\;\left(\%\right)=\;\frac{nº\;mammospheres}{nº\;seeded\;cells}\;\times \;100$$

### 2.9. ALDEFLUOR Assay

To analyze the aldehyde dehydrogenase (ALDH) activity, an ALDEFLUOR™ kit (Stem Cell Technologies, Durham, NC, USA) was used following the manufacturer indications. Cells were detached from the culture plastic (2D samples) and PCL scaffolds (3D) as explained in Section 2.8, washed with PBS and subsequently resuspended in ALDEFLUOR™ assay buffer at a concentration of 400,000 cells/mL. ALDEFLUOR™ Reagent (BODIPY-aminoacetaldehyde; BAAA) was added to each cell suspension. In order for the background fluorescence to be considered, a negative control for every sample was set by adding ALDEFLUOR™ diethylaminobenzaldehyde (DEAB), an ALDH inhibitor, to each cell suspension prior to adding BAAA in ALDEFLUOR assay buffer. All samples were incubated for 45 min at 37 °C in the dark.

Incubated samples were analyzed with a Cell Lab Quanta flow cytometer (Beckman Coulter Inc., Miami, FL, USA) to quantify the ALDH-positive cell population. The argon ion laser (488 nm) was used as a light source set at a power of 22 mW. Green fluorescence was detected with fluorescent channel 1 (FL1) optical filter (dichroic/splitter, dichroic long-pass: 550 nm, band-pass filter: 525 nm, detection width 505 to 545 nm). Information of a minimum of 10,000 events was recorded in List-mode Data files (LMD) and analyzed using FlowJo 10.2 software (FlowJo LLC, Ashland, OR, USA). Data were not compensated.

First, side-scatter (SS) and electronic volume (EV; equivalent to forward scatter) dot plots were performed and only single cells were selected, excluding debris and cells aggregates (less than 5%). Then, SS and log FL1 dot plots from DEAB samples were created to establish background fluorescence. The ALDH-positive cells’ gate was traced, delimiting the rightmost area and including only the 0.5% of total cell population. BAAA samples were equally processed and ALDH-positive cells gates of respective controls were used to discern the sample percentage of cells with high ALDH activity.

### 2.10. Statistical Analysis

All data are expressed as mean ± standard error of the mean (SEM). Data were analyzed using IBM SPSS (Version 21,0; SPSS Inc., Chicago, IL, USA). First, normality and homoscedasticity were evaluated using Shapiro-Wilks and Levene tests, respectively. As for cell proliferation and mammosphere forming assays, data were found to present a normal distribution and variances were homogeneous, a general linear model followed by post-hoc Sidak test was run. Factors were the treatment (i.e., 2D, 3D with 7.5% PCL, and 3D with 15% PCL and the culturing time). As far as the ALDEFLUOR assay, the same tests were run following transformation of data with arcsine square root (arcsin√*x*), as this was required for correcting the heteroscedasticity. Finally, as ratios between nuclear and cytoplasmic elongation factors (Fluorescence Microscopy Analysis in Section 3.3), did not fulfil with parametric assumptions, even when transformed, they were tested with Kruskal-Wallis and Mann-Whitney tests. The level of significance was set at *p* < 0.05. All observations were confirmed by at least three independent experiments.

## 3. Results

### 3.1. Electrospun Scaffolds Characterization

Once the polymer solution was electrospun, 7.5% PCL meshes showed an average thickness of 147.22 ± 5.00 µm, whereas 15% scaffolds’ depth was 196.00 ± 4.65 µm. To study microscopic scaffold architecture, both specimens were imaged by Scanning Electron Microscopy (SEM; Table 1). 

Meshes were visualized on the top and bottom sides so that fiber uniformity was certified, presenting similar features. Scaffolds from 7.5% PCL exhibited, apart from the filaments, spherical structures made by non-filamentous polymer. Regarding fiber diameter, 7.5% films showed an average diameter close to 300 nm, whereas the diameter of those containing 15% PCL increased up to 700 nm. Both scaffolds presented similar surface porosity, but they differed in the average pore area. Scaffolds from 15% PCL solution exhibited larger pores compared with 7.5% meshes.

### 3.2. Cell Proliferation

**Figure 1:**
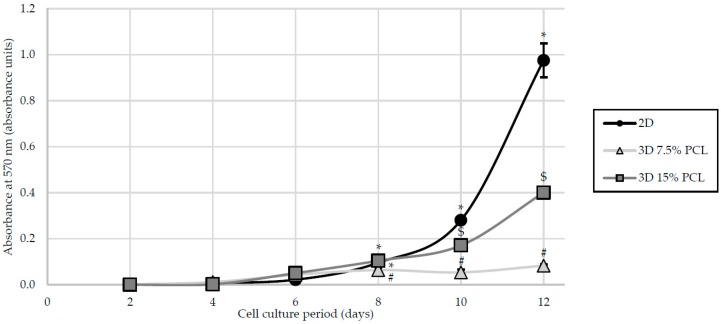
Cell proliferation analysis for MDA–MB–231 cells cultured on two-dimensional surfaces (2D) and on 7.5% and 15% PCL scaffolds (3D). (*), (#) and ($) symbols represent significant (*p* < 0.05) differences between groups.

As aforementioned, scaffolds could provide a three-dimensional environment for cancer cell culture. Architecture and porosity of filaments directly interfered with cell adhesion and growth. As seen in the previous section, 7.5% and 15% PCL scaffolds were proven to exhibit distinct microscopic structures. To evaluate the influence of scaffold microenvironment on cell growth, MDA–MB–231 cells were cultured on 2D adherent surfaces and on 7.5% and 15% meshes. Cell viability was evaluated through MTT assay on successive culture days, presented in Figure 1.

On the first assay days, few differences were observed regarding cell proliferation between different culture models. Variations between cell culture supports took place when a minimum cell confluence was reached, starting from day 8. MDA–MB–231 cells cultured on 2D adherent microplates presented a higher cell proliferation ratio compared to three-dimensional scaffolds, adopting strongly exponential kinetics. Scaffolds fabricated with 15% PCL solution also showed an exponential cell growth, but with a smaller slope. In contrast, 7.5% PCL meshes exhibited the lowest cell proliferation with a fairly linear trend. Since day 8, cell proliferation of 7.5% PCL scaffolds was significantly reduced when compared with monolayer culture (*p*-values ranging from <0.001 to 0.014) and 15% PLC meshes (*p*-values ranging from 0.002 to 0.040).

### 3.3. Cell Morphology

**Figure 2:**
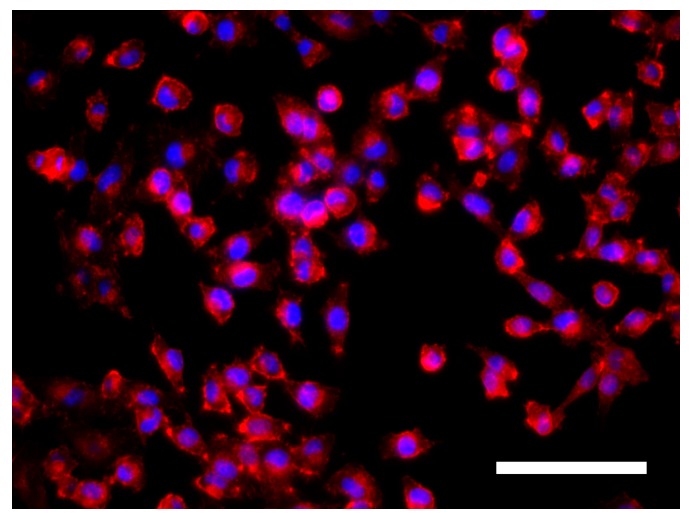
MDA–MB–231 cells grown in two-dimensional (2D) adherent coverslips. Actin cytoskeleton was stained with rhodamine-phalloidin (red) and nucleus was stained with 4,6-diamidino-2-phenylindole (DAPI; blue). Fluorescence microscopy images were captured at a magnification of 200× (Scale bar: 100 μm).

**Figure 3:**
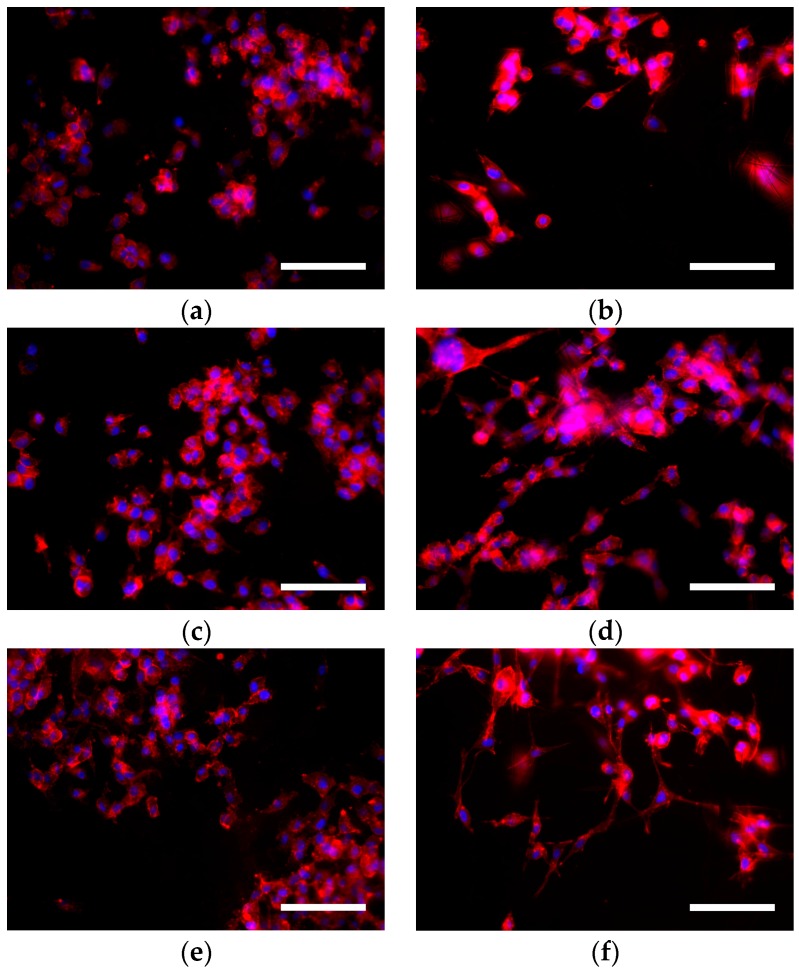
MDA–MB–231 cells grown in three-dimensional (3D) PCL scaffolds. Cells were seeded on 7.5% PCL (**a**,**c**,**e**) and 15% PCL meshes (**b**,**d**,**f**). Cells were cultured for 3 (**a**,**b**), 6 (**c**,**d**) and 12 days (**e**,**f**) without passage. Actin cytoskeleton was stained with rhodamine-phalloidin (red) and nucleus was stained with DAPI (blue). Fluorescence microscopy images were captured at a magnification of 200× (Scale bars: 100 µm).

MDA–MB–231 cells were cultured on adherent two-dimensional coverslips (2D) and three-dimensional PCL scaffolds (3D). Three different cell culture times (3, 6 and 12 days) were tested to evaluate whether morphology differences existed. Actin cytoskeleton and nucleus were stained to analyze possible changes in cell morphology between culture systems. MDA–MB–231 cells were routinely cultured on plastic cell culture dishes, establishing a cell monolayer where cells appeared to have a flattened structure. MDA–MD–231 cell line was also characterized to adopt a relatively lengthened cytoplasm. Fluorescent microscopy images confirmed the morphology described in 2D models (Figure 2). Some cells presented cytoplasmic prolongations, while others had a round shape and nucleus aspect was predominantly ellipsoidal. Then, MDA–MD–231 cells displayed different morphology when the two scaffold types, 7.5% and 15% PCL, were compared. Cells cultured on 7.5% PCL meshes (Figure 3a–c) exhibited similar aspects to 2D cultured ones, including nucleus and cytoplasm architecture. This trend was observed along the different days of cell culture with no noticeable differences. In contrast, a high number of MDA–MD–231 cells showed lengthened morphology when cultured on 15% PCL scaffolds (Figure 3d–f). Cytoplasm prolongations were longer than the ones from 2D and 3D 7.5% PCL cultures. When cell culture days increased, prolongations seemed to be even more extended. Moreover, some cells appeared to be unfocused on 15% PCL meshes pictures, indicating that cell culture occurred on different scaffold depth. No qualitative differences were observed concerning nuclear shape.

**Figure 4:**
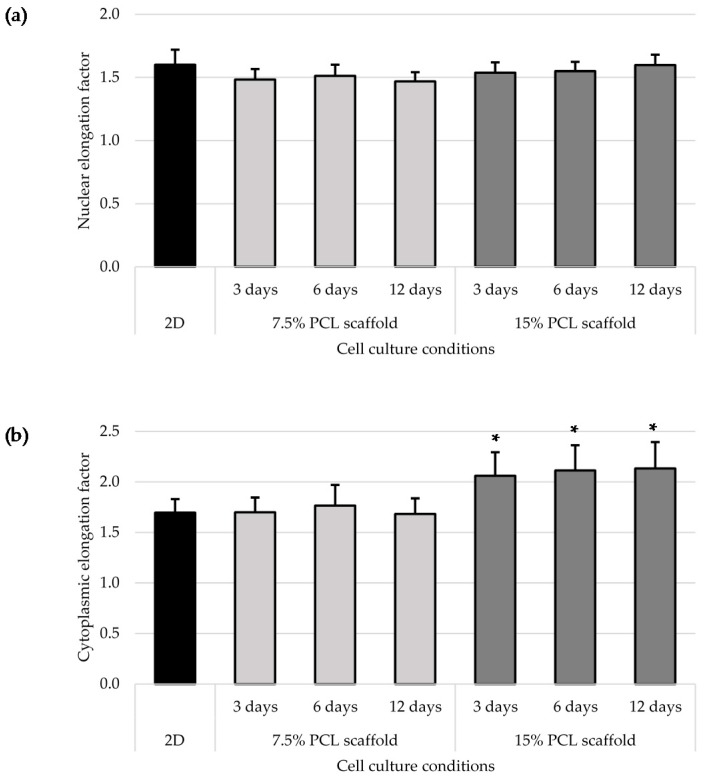
Cell morphology analysis for MDA–MB–231 cells cultured on two-dimensional surfaces (2D) and on 7.5% and 15% PCL scaffolds. Nuclear (**a**) and cytoplasmic elongation (**b**) were measured as explained in Section 2.7. Scaffolds values were compared with 2D culture and levels of statistically significance were indicated as * (*p* < 0.05).

To quantitatively evaluate cell morphology in 2D and 3D cultures, nuclear and cytoplasmic elongation were measured as described in Section 2.7. No significant changes in nucleus elongation were observed between 2D and 3D cultures (Figure 4a), which agreed with the aforementioned descriptive microscopic observation. The nuclear elongation factor of 2D cultured cells was 1.60 ± 0.12, pointing out to the ellipsoidal form of the nucleus. All 3D cultured cells factors were similar or slightly lower, between 1.47 ± 0.07 and 1.60 ± 0.08. Cytoplasm pattern was also studied (Figure 4b) and, whereas MDA–MB–231 cells cultured on 7.5% PCL scaffolds presented a similar cytoplasmic elongation factor than those cultured in 2D, with a value around 1.70, the cells on 15% PCL meshes exhibited a significantly higher cytoplasmic length.

As previously mentioned, SEM analysis showed that 15% PCL scaffolds were the only specimen formed exclusively by filaments. Moreover, MDA–MB–231 cells cultured on meshes of 15% PCL exhibited a high cell proliferation (Figure 1) and different cell morphology (Figure 3) compared with 7.5% PCL scaffolds. As the main aim of three-dimensional cell culture is to mimic the extracellular matrix structure and provide a comfortable support to cell growth, meshes from 15% PCL solution were chosen to conduct the further experiments of the present study. From now onwards, 3D culture samples will exclusively refer to cells cultured on 15% PCL scaffolds. As in previous experiments, the effects of culturing cells with those 15% PCL scaffolds were also tested at 3, 6 and 12 days of culture.

### 3.4. Mammoshperes Forming Assay

**Figure 5:**
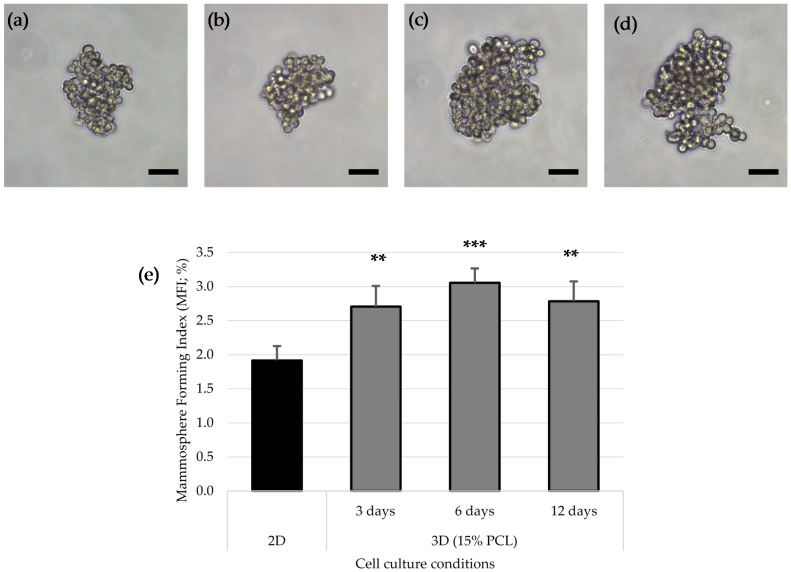
Mammosphere Forming Assay. Mammosphere images of MDA–MB–231 cells previously cultured on 2D surfaces (**a**) and 15% PCL scaffolds for 3 (**b**), 6 (**c**) and 12 days (**d**). (Scale bars: 50 μm). Mammosphere Forming Index (MFI) of MDA–MB–231 cell line after 2D or 3D cell culture with 15% PCL scaffolds (**e**). Significant differences of 3D with regard to 2D cultures are indicated as ** (*p* < 0.01) and *** (*p* < 0.001).

Breast cancer stem cells possess an anchorage-independent growth, proliferating into mammospheres when cultured on non-adherent surfaces. Hence, Mammospheres Forming Assay was first used to evaluate the spheres forming capacity of cells previously cultured on 2D and 3D supports. The MDA–MB–231 triple negative cell line was seeded as described in “Materials and Methods” section. Mammospheres from all cell samples did not show qualitative differences regarding morphology and size (Figure 5a–d). However, all three-dimensional cultured cells showed a significantly higher Mammosphere Forming Index (MFI) than 2D samples, reaching the maximum value and significance on 6 days of culture (Figure 5e).

### 3.5. Aldehyde Dehydrogenase Activity

**Figure 6:**
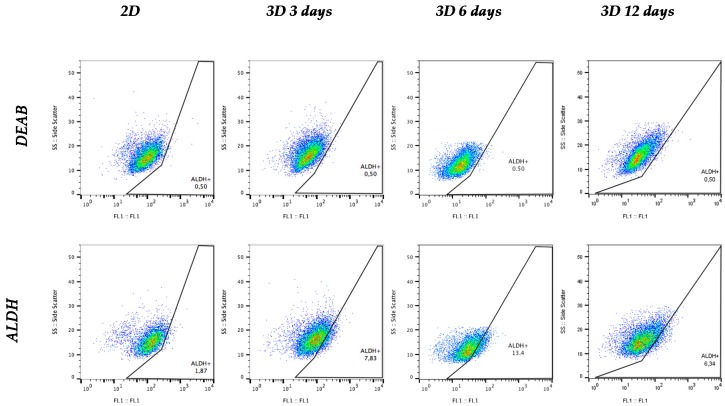
ALDEFLUOR assay plots of MDA–MB–231 cells cultured on monolayer (2D) and on 15% PCL scaffolds (3D). Aldehyde dehydrogenase (ALDH) inhibitor (diethylaminobenzaldehyde; DEAB) was processed to determine background fluorescence limit, gating 0.5% ALDH-positive cells for all samples.

The aldehyde dehydrogenase (ALDH) family is composed by dehydrogenases enzymes responsible for the oxidation of retinol (vitamin A) to retinoic acid [31], the latter of which is involved in gene expression regulation [32] and embryo development [33]. Moreover, ALDH enzymes have a detoxification role, protecting organisms against damaging aldehydes [34] and cytotoxic agents [35], and have also been demonstrated to regulate hematopoietic stem cells differentiation via retinoic acids production [36]. To corroborate the relative proportion of CSCs in 2D and 3D cultured cells, ALDH activity was quantified as a measure of stem properties. Samples were assessed with ALDEFLUOR assay and the percentage of ALDH-positive cells was determined as shown in Figure 6. 

**Figure 7:**
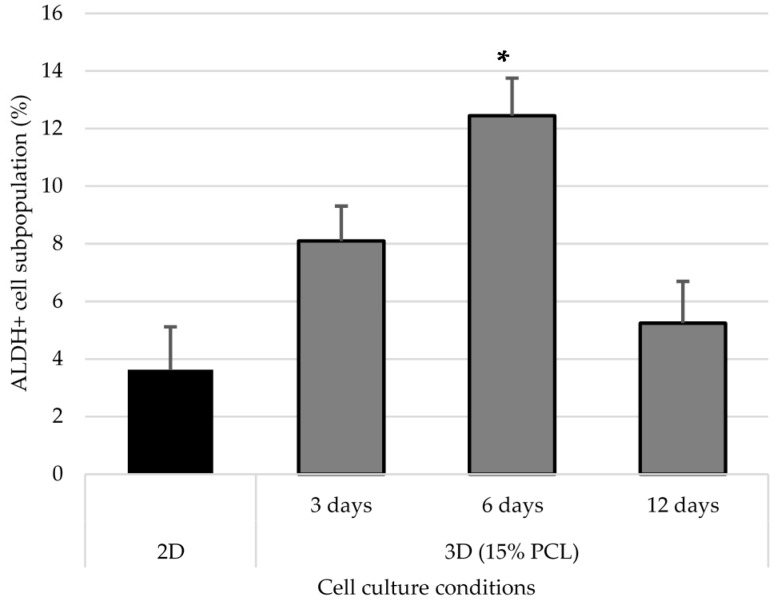
ALDH + cell subpopulation of MDA–MB–231 cells after monolayer culture (2D) or three-dimensional cell culture with 15% PCL scaffolds (3D). (*) Denotes significant (*p* < 0.05) differences between 3D culture at a given time point and 2D.

The ALDH-positive subpopulation increased when cells were cultured on PCL scaffolds, compared to monolayer culture (Figure 7). ALDH activity enhancement was observed at 3 and 6 days of cell culture, the latter time point being the one with a significant major percentage. However, the proportion of ALDH + cells after 12 days of culture in 15% PCL scaffolds decreased, with figures close to those of 2D.

## 4. Discussion

Polymer concentration (PCL) has been found to exert a great influence on scaffold architecture. Indeed, 7.5% PCL scaffolds showed polymeric spheres connected to filaments. These structures, previously described in the literature, are called beads and result from low polymer concentration [8,37]. In contrast, no beads were observed in 15% meshes, which also showed an average fiber diameter 2.4-fold higher than that of 7.5% specimens. While performing a direct comparison with values from other studies is difficult owing to the wide range of electrospun parameters used, the significant impact of PCL concentration in acetone on fiber morphology and diameter agrees with most previous studies [7,8,38,39]. Furthermore, pore parameters were also evaluated and, while electrospun meshes presented similar surface porosity, the average pore area in 15% scaffolds was 3.5-fold larger than that of 7.5% models.

MDA–MB–231 cells were successfully expanded on electrospun mats. Both scaffolds tested (i.e., 7.5% and 15%) showed less cell proliferation compared with the homologous monolayer culture, which was in agreement with previous studies [40]. When scaffolds from different polymer concentrations were compared, microarchitecture traits seemed to influence 3D cell culture efficiency. It is worth mentioning that low filament diameters have been revealed to facilitate cell adhesion and growth [38,41,42] and collagen fibers (the main protein in the extracellular matrix) display small diameters below 100 nm [43]. However, in the current study, the scaffold with the lowest fiber diameter (i.e., 7.5%) also exhibited beads which interfered with cell proliferation. Chen et al. (2007) demonstrated that, despite having smaller fiber diameters, meshes with beads reduce fibroblast growth [38]. In our conditions, beaded scaffolds exhibited a lower surface area-to-volume ratio, providing less material for cell growth. Moreover, interconnected pores with a minimum optimal area are needed to allow cell growth and scaffold infiltration. Small pores of 7.5% scaffolds may also hinder MDA–MB–231 cells’ penetration within the mesh structure. Following this hypothesis, most cells would have adhered to the surface and they would hardly have colonized different scaffold depths. With regard to cell morphology, MDA–MB–231 cells seeded on 15% PCL scaffolds presented more cytoplasmic elongations compared with round shaped cells on 7.5% PCL meshes and, specially, flat surfaces. This finding was supported by the higher cytoplasmic elongation factor of 15% PCL mat cells. Cell elongation along scaffold nanofilaments has also been observed in breast cancer cells [27,28], fibroblasts [44] and murine adult neural stem cells [45]. Accordingly, meshes from 15% PCL mimic the physiological environment better as they allow cells to set a structure that 2D monolayers are devoid of. In fact, cytoskeleton reorganization caused by 3D cell culture may regulate gene expression [2].

Taking into account cell proliferation kinetics and morphology changes, scaffold from 15% PCL solution was selected to further accommodate MDA–MB–231 cell culture and evaluate BCSCs niche expansion capacity. The MDA–MB–231 cell line presents low MFI values and can only be propagated few passages on suspension culture due to their moderate e-cadherin expression [46]. However, scaffolds culture improved mammospheres forming ability, particularly after 6 days of culture, resulting in an enlarged tumorigenic [47] and self-renewal potential [48]. Additionally, MDA–MB–231 cells cultured on electrospun mats over the same period showed a significant ALDH-positive population increase in comparison of standard culture, with a conclusive 3.4-fold increase. Greater ALDH activity indicated stem features acquisition since several studies noticed a high ALDH activity on mammary [19], hematopoietic [49] and leukemic stem cells [50]. Taking all described stem features assays into account, the present study has shown that 3D culture with electrospun scaffolds enhances triple negative MDA–MB–231 tumourigenicity and ALDH activity. These rearrangements reach the maximum significance when the culture period lasts 6 days, this time being the one that allows reaching the greatest BCSCs expansion through 3D cell culture. In agreement with our results, different breast cancer cell studies also demonstrated cancer stem cell amplification through PCL scaffolds fabricated by electrospinning [27,28,29] and by other methods such as additive manufacturing technologies [30].

In conclusion, the current study has revealed the vast potential of poly(ε-caprolactone) on the in vitro cell culture field. Electrospun PCL solutions resulted in nanofiber production with different architecture traits, demonstrating the high versatility of polymer and technology. Moreover, non-beaded PCL scaffolds have been proven to supply physical support for triple negative breast cancer cell proliferation and elongation. The 3D cell culture expanded the breast cancer stem cell subpopulation, which in turn expressed more malignancy markers and exhibited stem cell characteristics. Therefore, 3D culture with electrospun PCL nanofibers may be useful to maintain the in vivo structure and to culture BCSCs, making their expansion and characterization possible. Investigation of this rare subpopulation is much warranted as it could facilitate the development of new specific therapeutic approaches to prevent the high recurrence of tumors such as triple negative breast cancer. 

## Figures and Tables

**Table 1:** Microscopic characterization of 7.5% and 15% electrospun poly (ε-caprolactone) (PCL) scaffolds (7 kV, 6 mL/h). Top and bottom sides were visualized through scanning electron microscopy micrographs at different magnifications. Both sides were used to calculate fiber diameter, surface porosity and pore area. (Scale bars: 10 μm).

	Side	Magnification		
		1500×	5000×	
**7.5% PCL**	**Top**	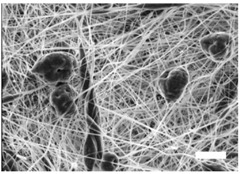	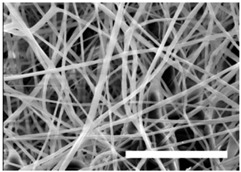	**Fibre diameter** 295.12 ± 148.45 nm **Surface porosity** 28.39% ± 4.53% **Pore area** 0.24 ± 0.42 µm
	**Bottom**	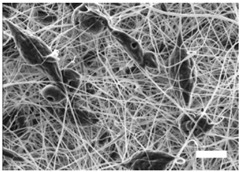	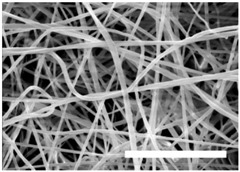	
**15% PCL**	**Top**	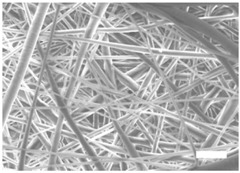	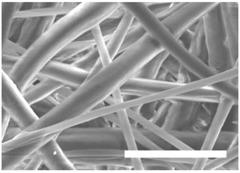	**Fibre diameter** 701.13 ± 401.89 nm **Surface porosity** 22.48% ± 7.57% **Pore area** 0.84 ± 1.82 µm^2^
	**Bottom**